# SARS-CoV-2 Superspread in Fitness Center, Hong Kong, China, March 2021

**DOI:** 10.3201/eid2708.210833

**Published:** 2021-08

**Authors:** Daniel K.W. Chu, Haogao Gu, Lydia D.J. Chang, Sammi S.Y. Cheuk, Shreya Gurung, Pavithra Krishnan, Daisy Y.M. Ng, Gigi Y.Z. Liu, Carrie K.C. Wan, Dominic N.C. Tsang, Malik Peiris, Leo L.M. Poon

**Affiliations:** The University of Hong Kong, Hong Kong, China (D.K.W. Chu, H. Gu, L.D.J. Chang, S.S.Y. Cheuk, S. Gurung, P. Krishnan, D.Y.M. Ng, G.Y.Z. Liu, C.K.C. Wan, M. Peiris, L.L.M. Poon);; The Government of Hong Kong Special Administrative Region, Hong Kong (D.N.C. Tsang)

**Keywords:** 2019 novel coronavirus disease, coronavirus disease, COVID-19, severe acute respiratory syndrome coronavirus 2, SARS-CoV-2, viruses, respiratory infections, zoonoses, superspread, transmission, molecular epidemiology, fitness center, Hong Kong, China

## Abstract

To investigate a superspreading event at a fitness center in Hong Kong, China, we used genomic sequencing to analyze 102 reverse transcription PCR–confirmed cases of severe acute respiratory syndrome coronavirus 2 infection. Our finding highlights the risk for virus transmission in confined spaces with poor ventilation and limited public health interventions.

Hong Kong, China, is at the end of a fourth wave of severe acute respiratory syndrome coronavirus 2 (SARS-CoV-2) infection. The virus causing this wave was introduced in September 2020 (GISAID clade GH) (*1*) and has continued to evolve in Hong Kong. As of April 30, 2021, a total of 11,771 SARS-CoV-2 cases had been laboratory confirmed; more than half (56%) were detected during the fourth wave. We describe a superspreading event that occurred in a 3,000-ft^2^ fitness center in March 2021 (Appendix Figure 1).

On March 10, 2021, an asymptomatic 27-year-old male fitness trainer (patient FC1) received a positive reverse transcription PCR (RT-PCR) test result as part of a voluntary coronavirus disease (COVID-19) screening program. This program provided services to persons for community or private purposes (e.g., for work or travel). The fitness trainer had previously received a negative COVID-19 test result on February 17, 2021. He taught small group classes in the fitness center every day from February 28 through March 8, except March 4, 2021. 

His positive test result triggered a local health authority to conduct epidemiologic investigation and contact tracing. The fitness center was immediately closed to the public. The local government also issued a compulsory testing notice to those who had visited this center from February 25 through March 10. About 300 visitors were tested and 101 cases were confirmed (7 staff members and 94 customers; case-patients FC2–FC102) (Appendix Table 1). All case-patients had recently visited this center; >80% of cases were detected within 3 days of the first case (Appendix Figure 2). Another 53 SARS-CoV-2–positive persons were subsequently identified; they had had close contact with the 102 case-patients but no epidemiologic link to the fitness center.

Of the 102 case-patients, all were hospitalized according to local standard practice, recovered uneventfully, and were discharged. None had received COVID-19 vaccination before this outbreak. A total of 46 case-patients were asymptomatic at the time of testing. The percentage of asymptomatic case-patients in this cluster (45%) is higher than that of all persons with confirmed cases in Hong Kong (30%; p<0.005). It is not known whether the general physical well-being of case-patients in this cluster affected their clinical status. Their ages, on average, were lower than that of all persons with confirmed cases in Hong Kong (38 vs. 44 years; p<0.005).

Among the 56 symptomatic case-patients, signs and symptoms started to develop for 36 of them during March 9–11; the earliest onset date was March 6 (case-patient FC46). Assuming the average incubation period of COVID-19 to be ≈5 days (*2*), the superspreading event might have occurred around March 5. Because SARS-CoV-2 can be transmitted by asymptomatic and presymptomatic persons (*3*), our data did not enable us to identify the index case-patient of this cluster.

To exclude unrelated transmission chains in this fitness center, we used next-generation sequencing to study respiratory samples from 59 of the case-patients (*1*,*4*). We used 5 epidemiologically unrelated local case-patients, including 4 detected in the same period, as controls. All virus sequences from the fitness center outbreak genetically clustered together and were genetically distinct from the controls (Figure), demonstrating that this superspreading event was caused by a single virus introduction.

**Figure:**
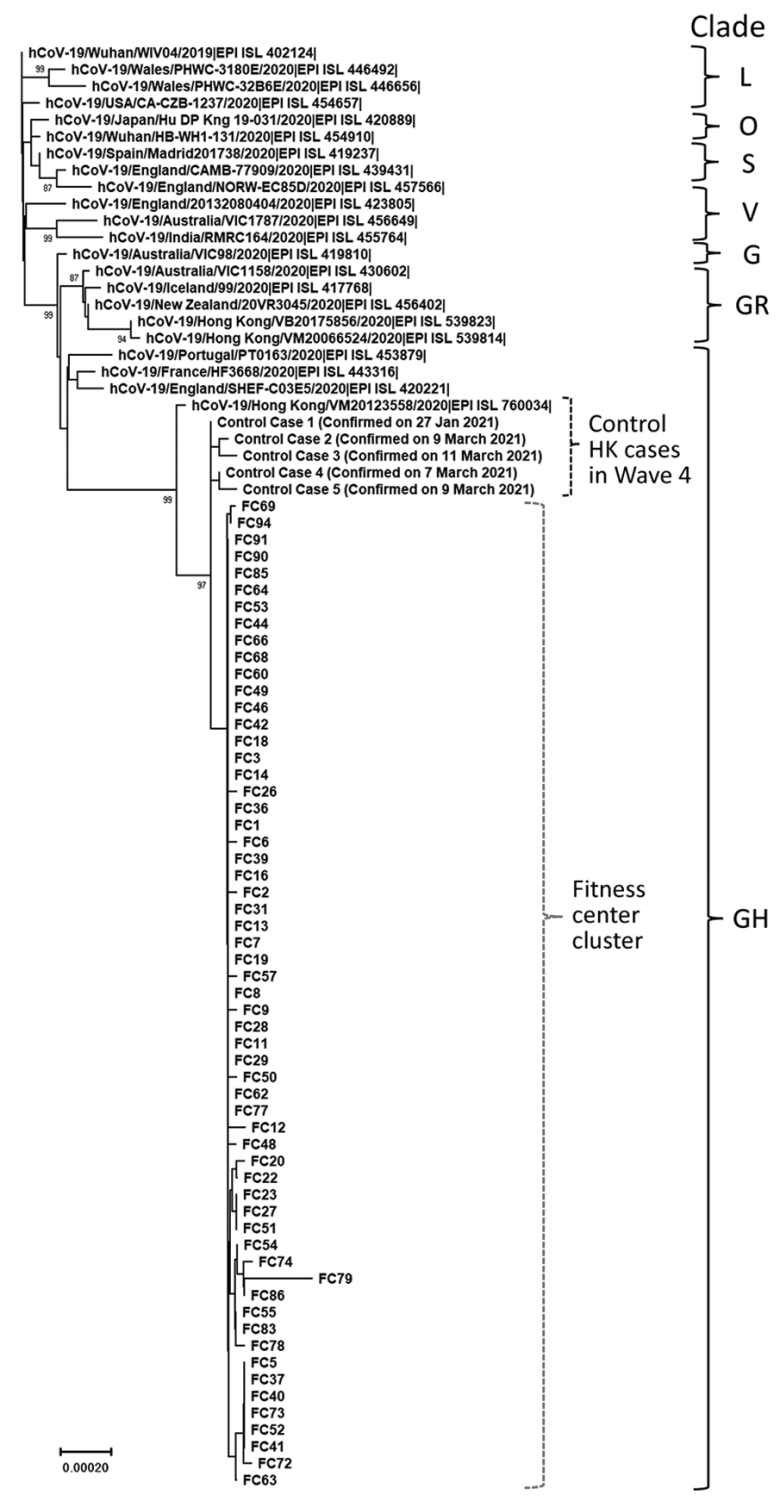
Phylogenetic tree of the severe acute respiratory syndrome coronavirus 2 (SARS-CoV-2) viruses detected in a fitness club in Hong Kong, China, in March 2021. Viruses from clades L, S, V, G, GH, GR, and O (others) are also included in the analysis. Near full-length genomes of studied samples were deduced by a previously described Illumina (https://www.illumina.com) sequencing protocol (sequence coverage >100) (*1**,**4*). Human SARS-CoV-2 WIV04 is selected to be the root of this phylogenetic tree. The tree was constructed by using the neighbor-joining method. Only bootstrap values >80 are shown. EPI ISL accession nos. for sequences retrieved in GISAID (https://www.gisaid.org) are provided. Scale bar indicates estimated genetic distance.

 Many case-patients, including FC1 and FC46, were located at the root of this phylogenetic branch. There are a few minor clades in this phylogenetic branch, suggesting that the initial introduction triggered multiple independent transmission chains thereafter in this setting.

SARS-CoV-2 transmission in fitness centers/gyms has been reported (*5*–*8*). SARS-CoV-2 can be transmitted by close contact, droplets, or fomites (*9*). Uncontrolled physical activities in a fitness center might produce any or all of these transmission modes (e.g., increased physical contact, increased levels of exhaled respiratory droplets in a confined space because of vigorous breathing, and shared communal space and equipment). Although in this study we were unable to identify the predominant transmission mode accounting for this superspreading event, a recent report indicates that physical activities in a fitness center can create a pronounced level of saliva aerosol (*10*). An air change rate of 2.2/hour in a fitness center is insufficient to dilute the amount of saliva aerosol generated from physical activities (*10*). Of note, mask wearing during exercise was not compulsory by law at the time of this outbreak. Many case-patients in our study reported not wearing a mask while training at that time (e.g., weight training, high-intensity circuit training, and boxing). A follow-up investigation revealed that this center has air conditioning units but lacks a fresh air and exhaust duct system. This finding suggests that poor ventilation might have played a major role in this outbreak.

After this outbreak, new recommendations were issued to prevent superspreading events at fitness centers in Hong Kong. For staff in these settings, RT-PCR testing for SARS-CoV-2 every 2 weeks is compulsory, and staff are prioritized to receive COVID-19 vaccination. For all persons in fitness settings, mask wearing at all times is now mandatory, except when showering or eating. Recommendations for air ventilation in all fitness centers are under investigation.

Appendix Supplementary data for study of SARS-CoV-2 superspread in fitness center, Hong Kong, China, March 2021.
