# Supplementary material for: SARS-CoV-2 Superspread in Fitness Center, Hong Kong, China, March 2021
Source: Emerg Infect Dis. 2021 Aug;27(8):2230–2. doi: 10.3201/eid2708.210833 (PMC8314845; doi:10.3201/eid2708.210833)
Supplement: Appendix — Supplementary data for study of SARS-CoV-2 superspread in fitness center, Hong Kong, China, March 2021. [file 21-0833-Techapp-s1.pdf]

Article DOI: https://doi.org/10.3201/eid2708.210823

SARS-CoV-2 Superspread in Fitness Center, Hong Kong, China, March 2021

Appendix

Appendix Table 1. Epidemiology data of FC1-FC2

Case no.	Report date (2021)	Sex	Age	Onset date (2021)
FC1	Mar 10	Male	27	Asymptomatic
FC2	Mar 11	Male	46	Mar 9
FC3	Mar 11	Male	40	Asymptomatic
FC4	Mar 11	Male	56	Asymptomatic
FC5	Mar 11	Male	30	Mar 8
FC6	Mar 11	Male	44	Mar 8
FC7	Mar 11	Male	47	Mar 9
FC8	Mar 11	Female	23	Mar 8
FC9	Mar 11	Female	30	Mar 9
FC10	Mar 11	Female	30	Mar 9
FC11	Mar 11	Male	34	Asymptomatic
FC12	Mar 11	Male	41	Asymptomatic
FC13	Mar 11	Female	19	Mar 9
FC14	Mar 11	Male	47	Mar 9
FC15	Mar 11	Female	54	Asymptomatic
FC16	Mar 11	Female	31	Asymptomatic
FC17	Mar 11	Male	35	Asymptomatic
FC18	Mar 12	Female	42	Mar 8
FC19	Mar 12	Male	34	Mar 7
FC20	Mar 12	Female	42	Asymptomatic
FC21	Mar 12	Male	39	Asymptomatic
FC22	Mar 12	Male	33	Mar 10
FC23	Mar 12	Female	26	Mar 10
FC24	Mar 12	Male	36	Mar 9
FC25	Mar 12	Male	34	Mar 10
FC26	Mar 12	Male	39	Mar 10
FC27	Mar 12	Male	53	Mar 10
FC28	Mar 12	Female	33	Asymptomatic
FC29	Mar 12	Male	42	Mar 11
FC30	Mar 12	Male	37	Asymptomatic
FC31	Mar 12	Male	46	Mar 10
FC32	Mar 12	Female	38	Mar 9
FC33	Mar 12	Male	41	Asymptomatic
FC34	Mar 12	Female	22	Asymptomatic
FC35	Mar 12	Male	36	Asymptomatic
FC36	Mar 12	Male	20	Asymptomatic
FC37	Mar 12	Female	35	Mar 10
FC38	Mar 12	Female	33	Mar 10
FC39	Mar 12	Female	24	Mar 10
FC40	Mar 12	Female	37	Mar 11
FC41	Mar 12	Male	36	Mar 11
FC42	Mar 12	Female	38	Asymptomatic
FC43	Mar 12	Male	40	Mar 9
FC44	Mar 12	Female	36	Mar 9
FC45	Mar 12	Male	44	Mar 8
FC46	Mar 12	Female	32	Mar 6
FC47	Mar 12	Male	34	Asymptomatic
FC48	Mar 12	Female	37	Mar 10
FC49	Mar 12	Male	41	Mar 9
FC50	Mar 12	Male	27	Asymptomatic
FC51	Mar 12	Male	45	Asymptomatic
FC52	Mar 12	Male	31	Mar 11
FC53	Mar 12	Female	44	Asymptomatic
FC54	Mar 12	Female	25	Asymptomatic

Case no.	Report date (2021)	Sex	Age	Onset date (2021)
FC55	Mar 12	Female	38	Asymptomatic
FC56	Mar 12	Male	41	Mar 11
FC57	Mar 12	Male	36	Asymptomatic
FC58	Mar 13	Female	37	Mar 11
FC59	Mar 13	Female	37	Asymptomatic
FC60	Mar 13	Male	61	Asymptomatic
FC61	Mar 13	Female	28	Mar 11
FC62	Mar 13	Male	48	Asymptomatic
FC63	Mar 13	Female	34	Mar 11
FC64	Mar 13	Male	36	Mar 11
FC65	Mar 13	Female	31	Mar 11
FC66	Mar 13	Male	35	Mar 10
FC67	Mar 13	Female	36	Mar 11
FC68	Mar 13	Male	40	Asymptomatic
FC69	Mar 13	Male	49	Mar 12
FC70	Mar 13	Male	33	Asymptomatic
FC71	Mar 13	Male	38	Asymptomatic
FC72	Mar 13	Male	37	Asymptomatic
FC73	Mar 13	Male	48	Mar 11
FC74	Mar 13	Male	62	Asymptomatic
FC75	Mar 13	Male	39	Asymptomatic
FC76	Mar 13	Male	36	Asymptomatic
FC77	Mar 13	Male	33	Mar 12
FC78	Mar 13	Female	36	Mar 12
FC79	Mar 13	Female	48	Asymptomatic
FC80	Mar 13	Male	26	Mar 9
FC81	Mar 13	Female	36	Asymptomatic
FC82	Mar 13	Female	39	Mar 10
FC83	Mar 14	Male	47	Mar 13
FC84	Mar 14	Female	37	Asymptomatic
FC85	Mar 14	Male	31	Asymptomatic
FC86	Mar 14	Male	46	Asymptomatic
FC87	Mar 14	Female	34	Mar 13
FC88	Mar 14	Female	37	Mar 13
FC89	Mar 15	Female	43	Asymptomatic
FC90	Mar 15	Male	47	Mar 13
FC91	Mar 15	Female	45	Mar 13
FC92	Mar 15	Female	36	Mar 14
FC93	Mar 16	Male	42	Asymptomatic
FC94	Mar 17	Female	34	Asymptomatic
FC95	Mar 18	Male	42	Mar 16
FC96	Mar 19	Male	46	Mar 17
FC97	Mar 20	Male	44	Asymptomatic
FC98	Mar 21	Female	37	Asymptomatic
FC99	Mar 22	Male	39	Mar 20
FC100	Mar 22	Female	28	Asymptomatic
FC101	Mar 22	Female	39	Mar 20
FC102	Mar 23	Male	33	Asymptomatic

Appendix Table 2. Studied sequences from GISAID

Virus name	Accession No.	Collected	Originating laboratory	Submitting laboratory	Submitted by
Australia/VIC1158/2020	EPI_ISL_430602	2020 Apr 9	Victorian Infectious Diseases Reference Laboratory (VIDRL)	Microbiological Diagnostic Unit Public Health Laboratory and Victorian Infectious Diseases Reference Laboratory, The Peter Doherty Institute for Infection and Immunity	Caly L. et al
Australia/VIC1787/2020	EPI_ISL_456649	2020 May 27	Victorian Infectious Diseases Reference Laboratory (VIDRL)	Microbiological Diagnostic Unit Public Health Laboratory and Victorian Infectious Diseases Reference Laboratory, Doherty Institute	Caly L. et al
Australia/VIC98/2020	EPI_ISL_419810	2020 Mar 16	Victorian Infectious Diseases Reference Laboratory (VIDRL)	Victorian Infectious Diseases Reference Laboratory and Microbiological Diagnostic Unit Public Health Laboratory, Doherty Institute	Caly L. et al
England/20132080404/2020	EPI_ISL_423805	2020 Mar 24	Respiratory Virus Unit, Microbiology Services Colindale, Public Health England	Respiratory Virus Unit, Microbiology Services Colindale, Public Health England	Monica Galiano et al
England/CAMB-77909/2020	EPI_ISL_439431	2020 Mar 31	Department of Pathology, University of Cambridge	Wellcome Sanger Institute for the COVID-19 Genomics UK (COG-UK) consortium	Luke W Meredith et al
England/NORW-EC85D/2020	EPI_ISL_457566	2020 May 13	Quadram Institute Bioscience	COVID-19 Genomics UK (COG-UK) Consortium	Dave J. Baker et al
England/SHEF-C03E5/2020	EPI_ISL_420221	2020 Mar 29	Virology Department, Sheffield Teaching Hospitals NHS Foundation Trust	Department of Infection, Immunity and Cardiovascular Disease, The Florey Institute, The Medical School, University of Sheffield	Thushan de Silva et al
France/HDF-3668/2020	EPI_ISL_443316	2020 Mar 25	CH Compiègne Laboratoire de Biologie	National Reference Center for Viruses of Respiratory Infections, Institut Pasteur, Paris	Mélanie Albert et al
HongKong/VB20175856/2020	EPI_ISL_539823	2020 Aug 14	Communicable Disease Branch	Hong Kong Department of Health	Alan K.L. Tsang et al
HongKong/VM20066524/2020	EPI_ISL_539814	2020 Jul 21	Tuen Mun Hospital	Hong Kong Department of Health	Alan K.L. Tsang et al
Iceland/99/2020	EPI_ISL_417768	2020 Mar 10	The National University Hospital of Iceland	deCODE genetics	Daniel F Gudbjartsson et al
India/OR-RMRC164/2020	EPI_ISL_455764	2020 May 7	REGIONAL VRDL,ICMR-RMRC BBSR	Immunogenomics lab, Institute of Life Sciences, Bhubaneswar	Sunil Raghav et al
Japan/Hu_DP_Kng_19-031/2020	EPI_ISL_420889	2020 Feb 14	Takayuki Hishiki Kanagawa Prefectural Institute of Public Health	Takayuki Hishiki Kanagawa Prefectural Institute of Public Health	Hishiki et al
NewZealand/20VR3045/2020	EPI_ISL_456402	2020 Apr 25	Wellington SCL	Institute of Environmental Science and Research (ESR)	Matt Storey et al

Virus name	Accession No.	Collected	Originating laboratory	Submitting laboratory	Submitted by
Portugal/PT0163/2020	EPI_ISL_453879	2020 Mar 28	unknown	Instituto Nacional de Saude (INSA)	Borges et al et al
Spain/MD-ISCIII-201738/2020	EPI_ISL_419237	2020 Mar 7	Fundacion Jimenez Diaz	Instituto de Salud Carlos III	Iglesias-Caballero et al
USA/CA-CZB-1237/2020	EPI_ISL_454657	2020 May 12	County of Santa Clara Public Health Department	Chan-Zuckerberg Biohub	CZB Cliahub Consortium et al
Wales/PHWC-3180E/2020	EPI_ISL_446492	2020 Apr 11	Wales Specialist Virology Centre	Public Health Wales Microbiology Cardiff	Catherine Moore et al
Wales/PHWC-32B6E/2020	EPI_ISL_446656	2020 Apr 14	Wales Specialist Virology Centre	Public Health Wales Microbiology Cardiff	Catherine Moore et al
Wuhan/HB-WH1-131/2020	EPI_ISL_454910	2020 Mar 2	Wuhan Chain Medical Labs (CMLabs)	State Key Laboratory of Biotherapy of Sichuan University	Baowen Du et al
Wuhan/WIV04/2019	EPI_ISL_402124	2019 Dec 30	Wuhan Jinyintan Hospital	Wuhan Institute of Virology, Chinese Academy of Sciences	Peng Zhou et al

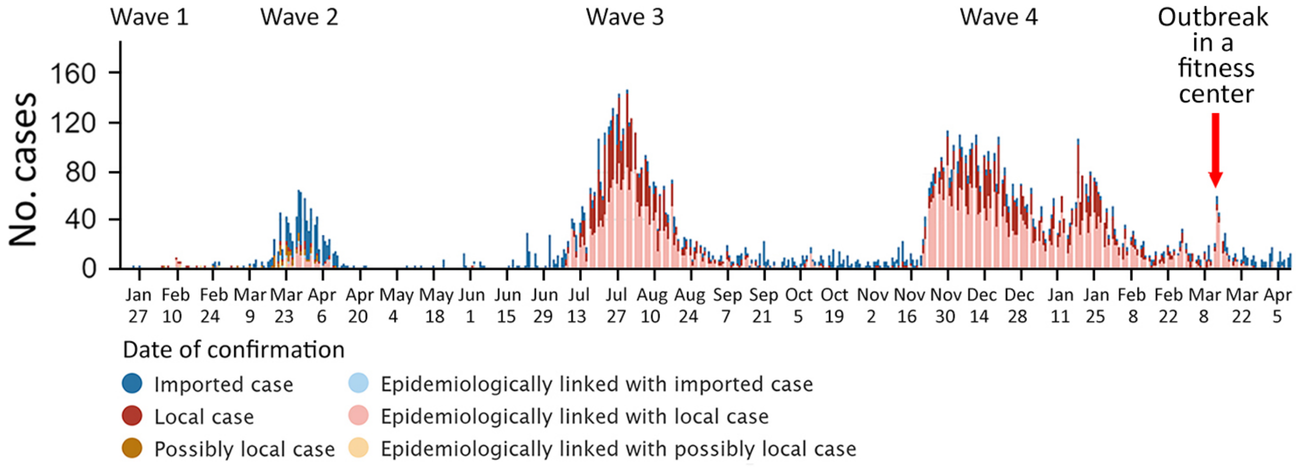

Appendix Figure 1. Coronavirus disease (COVID-19) pandemic waves in Hong Kong, China. Number of cases with different epidemiologic links are shown. Arrow indicates the outbreak that occurred in the fitness center in Hong Kong in March 2021. This Figure is modified from the one available from the School of Public Health, The University of Hong Kong (https://covid19.sph.hku.hk/).

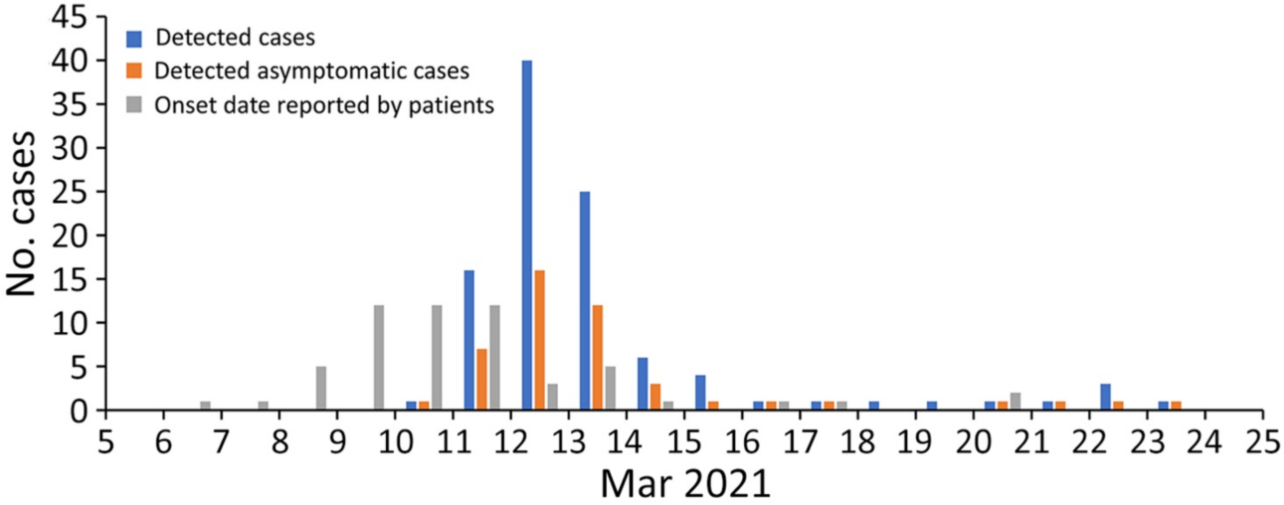

Appendix Figure 2. COVID-19 cases epidemiologically linked to a fitness center. The number of daily detected cases, daily detected asymptomatic cases, and reported onset dates in the studied period are shown. The first reported case was detected on 10 Mar 2021.
